# Oxidative Stress and Low-Grade Inflammation in Polycystic Ovary Syndrome: Controversies and New Insights

**DOI:** 10.3390/ijms22041667

**Published:** 2021-02-07

**Authors:** Antonio Mancini, Carmine Bruno, Edoardo Vergani, Claudia d’Abate, Elena Giacchi, Andrea Silvestrini

**Affiliations:** 1 Dipartimento di Medicina e Chirurgia Traslazionale, Università Cattolica del Sacro Cuore, 00168 Rome, Italy; carmine.bruno@outlook.it (C.B.); edoardo.vergani@outlook.it (E.V.); claudiadabate94@gmail.com (C.d.); 2 Fondazione Policlinico Universitario a Gemelli IRCCS, 00168 Rome, Italy; 3 Centro Studi e Ricerche per la Regolazione Naturale della Fertilità, Università Cattolica del Sacro Cuore, 00168 Rome, Italy; elena.giacchi@unicatt.it; 4 Dipartimento di Scienze Biotecnologiche di Base, Cliniche Intensivologiche e Peri-Operatorie, Università Cattolica del Sacro Cuore, 00168 Rome, Italy

**Keywords:** obesity, insulin-resistance, hyperandrogenism, antioxidants

## Abstract

The pathophysiology of Polycystic Ovary Syndrome (PCOS) is quite complex and different mechanisms could contribute to hyperandrogenism and anovulation, which are the main features of the syndrome. Obesity and insulin-resistance are claimed as the principal factors contributing to the clinical presentation; in normal weight PCOS either, increased visceral adipose tissue has been described. However, their role is still debated, as debated are the biochemical markers linked to obesity per se. Oxidative stress (OS) and low-grade inflammation (LGI) have recently been a matter of researcher attention; they can influence each other in a reciprocal vicious cycle. In this review, we summarize the main mechanism of radical generation and the link with LGI. Furthermore, we discuss papers in favor or against the role of obesity as the first pathogenetic factor, and show how OS itself, on the contrary, can induce obesity and insulin resistance; in particular, the role of GH-IGF-1 axis is highlighted. Finally, the possible consequences on vitamin D synthesis and activation on the immune system are briefly discussed. This review intends to underline the key role of oxidative stress and low-grade inflammation in the physiopathology of PCOS, they can cause or worsen obesity, insulin-resistance, vitamin D deficiency, and immune dyscrasia, suggesting an inverse interaction to what is usually considered.

## 1. Introduction

Polycystic ovary syndrome (PCOS) is a gynecological endocrine disorder afflicting female of fertile age. PCOS has always attracted researcher attention due to high incidence; since it is the most frequent endocrine/metabolic disorder in the female population (about 6–14% of childbearing age) [1] and cardiovascular/oncological risk in the affected cohort of patients. Its diagnosis is based on Rotterdam criteria [2] since hyperandrogenism, chronic oligo- or anovulation, and echographic pattern of polycystic ovaries are the main features, differently combined in the various phenotypes of the syndrome [3]. The role of hyperandrogenism is stressed for its relation to metabolic derangement; therefore, the AE-PCOS Society maintains hyperandrogenism as mandatory for diagnosis, coupled with chronic oligo-anovulation or polycystic ovaries [4]. It has been also proposed that PCOS without hyperandrogenism should be excluded from the syndrome as they are based on different etiologies [5].

**Figure 1:**
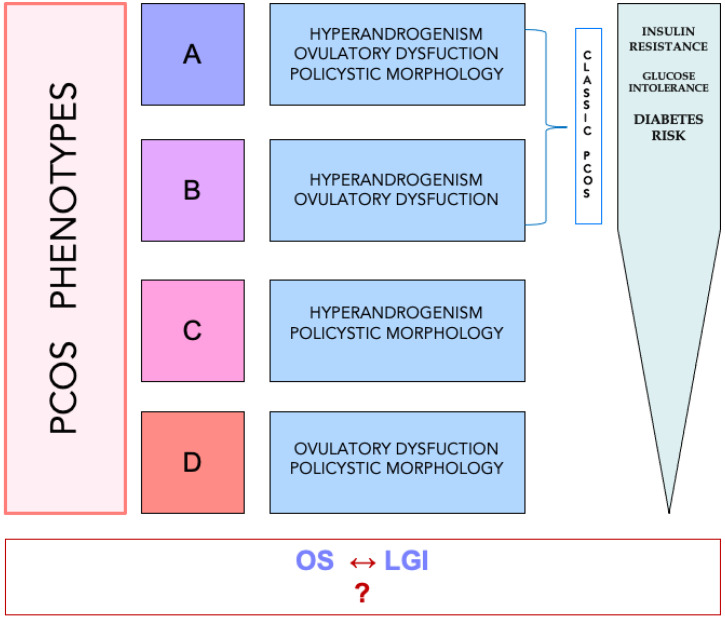
Diagram representing the classification of the different phenotypes of PCOS. While there is agreement on metabolic risk, which is higher in A-B phenotypes than C-D ones, clear correlation between phenotypes and parameters of oxidative stress (OS) and/or indexes of low-grade inflammation (LGI) is not defined (?).

The different criteria used for diagnosis can have consequences on epidemiological and clinical studies; moreover, different geographical areas can show a different prevalence of the syndrome, according to different diagnostic parameters. Therefore, in 2012 National Institute of Health (NIH) [6] recommended using a phenotypical classification, previously proposed by Azziz et al. [5], in four classes: (1) phenotype A, characterized by clinical or biochemical hyperandrogenism (HY), ovulatory dysfunction (OD), and polycystic ovarian morphology (PCOM); (2) phenotype B, presenting with hyperandrogenism (HA) and OD, but not PCOM; (3) phenotype C, showing HA and PCOM, without OD; and finally (4) phenotype D, including OD and PCOM, without HA. The phenotypes A and B are also called “classic PCOS”, the phenotype C is also indicated as “ovulatory PCOS”, while the last can be also marked as “non-hyperandrogenic PCOS”. The classical forms are the most prevalent. Nevertheless, some criticism has also been advanced to this classification, since most studies consider clinically evident subjects while the prevalence could be different in the general population [7]. Whatever the case, this classification, other than its clinical and statistical usefulness, also covers pathophysiological meaning, since it is generally accepted that phenotypes A and B present the higher prevalence of insulin resistance (IR) and risk to develop glucose intolerance or type 2 diabetes [8]. Figure 1 shows a diagram illustrating these concepts.

Even though extensive clinical data are published (combining “PCOS” and “physiopathology” keywords on Medline generates 3537 results, 1393 in the last ten years), many aspects remain unclear. The roles of visceral obesity, IR, hyperandrogenism overlap each other, with progressive vicious circles, making it difficult to design a univocal etiopathogenetic sequence. Based on the above-described phenotype classification, different studies have tried to correlate metabolic and hormonal parameters, without reaching an unequivocal picture: some studies underlined the risk of metabolic syndrome in hyperandrogenic phenotypes [9,10,11] while others pointed to the role of BMI [12] and total or abdominal obesity [13]. Regardless of obesity and IR, hyperandrogenic PCOS have a higher risk of liver steatosis [14]. Finally, it has been also reported that no significant difference in IR, glucose intolerance, and metabolic syndrome can be detected in the four groups [15]. Interestingly, a cohort of a specific geographical area (Korea) mainly included young non-obese women [16], strengthening the prevalence of different clinical phenotypes in different clinical areas.

Indeed, it is clear that oxidative stress (OS) is involved in PCOS disorder. Modern concepts concern the presence of low-grade inflammation (LGI) and the contribution of oxidative stress (OS). OS can be defined as “a disturbance in the prooxidant/antioxidant balance in favor of the former, potentially leading to damage” [17]. OS discloses when the levels of oxidants significantly overcome the antioxidant defenses. Notably, the initial view of oxidative stress has focused on the pathological role of reactive oxygen species (ROS) in the development and progression of the major human diseases (e.g., cancer, diabetes, atherosclerosis, cardiovascular diseases). It has been assessed that OS is associated with over a hundred diseases, either as their cause or consequence [18]. However, increasing evidences have highlighted that ROS are not just harmful agents implicated in the pathogenesis of various disorders and diseases. ROS can also act as mobile redox messengers in the regulation of numerous signaling pathways ranging from cell homeostasis to cell death [19,20] as reported in the ovarian physiology, as we previously reviewed [21]. Consequently, the idea that “ROS are unfavorable, and antioxidants are favorable” has undergone a critical and considerable reappraisal. Accordingly, ROS can act as a second messenger, regulate gene expression, and consequently influence cell growth, differentiation, and apoptosis. Only one study evaluated the impact of different phenotypes on OS [22]: once again patients with HA presented an increased OS suggested by increased antioxidant status measured by a colorimetric method. Therefore, this field still remains open to be investigated.

Chronic low-grade inflammation syndromes are systemic and chronic pathological conditions characterized by a slight increase in inflammatory markers; PCOS has been considered part of this group. Chronic LGI exhibits increased circulating cytokine levels and macrophage infiltration in peripheral tissues, which do not cause, however, any damage or loss of function to the involved tissues [23,24,25,26]. According to Margioris et al., high sensitivity C-reactive protein (CRP) is considered the best marker to be measured for clinical purposes: levels <1 mg/L suggest no chronic LGI; levels between 1 and 3 mg/L suggests clinically evident chronic LGI; levels between 3 and 10 mg/L suggests a high chronic LGI and levels higher than 10 mg/L suggest acute inflammation not related to chronic LGI [27]. Both antioxidant and anti-inflammatory therapies have been therefore proposed [28,29,30].

On the other hand, prevalence of obesity ranges from 30 to 75% in different reports [31,32]. Even in absence of obesity, an increased percentage of body weight and central adiposity [33] has been detected. Body composition has been related to IR, which is described also in normal-weight PCOS (NW-PCOS). Therefore, this point has been considered to play a key role in the development of the syndrome. 

One of the aims of the present paper is to review studies about OS and inflammatory markers in PCOS and evidence in favor or against the role of obesity as a pathogenetic starting point of vicious circles of the syndrome.

## 2. Mechanism of Oxidative Stress in PCOS

Both genetic and environmental factors can exacerbate OS. 

Genetic studies have concerned genes involved in steroid synthesis, gonadotropin receptors, IR [34]. Mitochondrial dysfunction has been addressed as a central phenomenon since mitochondria carry out a pivotal role in cell energy mechanisms, representing the main source of ROS as by-products of nutrient translation [35]. An increased ROS production can induce damage of mitochondrial components such as mtDNA, proteins, lipids and finally prompts cell apoptosis mediated by mitochondrial alterations [36]. The abnormalities of the mitochondrial genome have been recently revised [35]. Two main aspects must be considered. The first one is the number of mtDNA copies, which have been found to be lower in PCOS [37], with a negative correlation with the severity of the syndrome. The alteration of mtDNA copies is crucial for the ROS increase [38]. The second aspect is the discovery of mitochondrial gene mutation. Some investigators found single point mutations of genes encoding mitochondrial transfer RNA (mt-tRNA) associated with metabolic complications of PCOS, such as diabetes and hypertension [39,40]. Single-nucleotide polymorphism of a non-coding region of mtDNA (D-loop) has been reported in a cohort of south Indian women [41]. Finally, other genes involved in mitochondrial oxidative metabolism have been possibly related to PCOS development [42,43]. 

Diet-induced OS: glucose absorption induces an inflammatory response as documented by increased ROS-related OS and increased NFkB activation that is independent of obesity [44,45]. The release of tumor necrosis factor (TNF)α and IL-6 from circulating monocytes, induced by glucose ingestion, is altered in PCOS [46], and confirming data are reported in vitro [47]. These markers of OS and LGI are associated with index of insulin sensitivity after load or fasting index of IR [44,45,47]. ROS generation and p47^phox^ protein (which translocates from cytosol to membrane, activating membrane-bound NADPH oxidase with generation of superoxide) increase has been reported also in normal-weight patients without increased abdominal fat [3]. Therefore, diet-induced response could contribute to IR.

Hormonal mechanisms: hyperandrogenemia can underlie diet-induced inflammatory response; indeed, the administration of oral androgens, rising circulating levels at values comparable to PCOS, induces, both in fasting state and after glucose load, mononuclear cell (MNC) activation, with consequent cascade of previously described events (i.e., ROS generation, NFkB activation and increase in TNFα mRNA). Androgens exert these actions via their receptor, as demonstrated by androgen-resistant mice models which were not influenced by testosterone (T) or dihydrotestosterone (DHT) treatment [48]. The above-cited study of Zhang [22] showed an increased total antioxidant status in PCOS patients with HA; main predictors of total antioxidant status in multivariate analysis were apolipoprotein (apo)A1, the Ferriman–Gallwey score, triglycerides, estradiol, high-density lipoprotein cholesterol and 2-h glucose levels. 

Early hyperandrogenism during fetal life in animal models has been related to PCOS, influencing balance between prenatal adipogenesis and lipogenesis [49]; it could induce a mismatch between subcutaneous and visceral adipose tissue. As underpinning mechanisms, low levels of adiponectin, and increased fatty acid levels, magnified by both genetic and epigenetic factors, lead to decreased storage capacity and ectopic fat deposition (liver, visceral adipose tissue). Adiponectin decrease, due to the key anti-inflammatory and insulin-sensitizing effect of the hormone, contributes to IR starting from childhood [49,50,51]; furthermore, during adulthood, upregulation of fat promoting genes in visceral adipose tissue could ensue. Recently animal models as a tool for understanding physiopathology of PCOS confirmed such view [52].

Moreover, other mechanisms could be related to the alteration of GH-IGF-1 axis (see below). 

OS can induce and/or worsen all main features of PCOS.

Insulin resistance: OS alters the glucose uptake in muscle and adipose tissue and reduces insulin secretion by pancreatic b-cells [53,54,55]. IR can be induced by intracellular signaling in response to OS, both in vitro [56,57,58] and in vivo [59,60]. Moreover, antioxidant treatments may improve insulin sensitivity in patients with IR or type 2 diabetes [61]. 

Obesity: increased OS induces obesity-promoting preadipocyte proliferation and adipocyte differentiation, by increasing the size of mature adipocytes [62]; a central effect has also been hypothesized since ROS can influence neurons regulating feeding behavior in favor of those inducing hunger [63]. 

Hyperandrogenism: infiltration of the ovary by MNC-derived macrophages has been described [64], CYP17 is upregulated by proinflammatory stimuli and inhibited by resveratrol [65]; TNFα can stimulate in vitro proliferation of theca cells which synthetize androgens [29]. The effects of hyperandrogenism are not surprising, due to the reports of prooxidant effects of T, both in vivo and in vitro, and the same ROS production caused the synthesis of androgens themselves [66].

Follicular apoptosis: while ROS have a positive role in inducing the completion of meiosis I in dominant follicle, antioxidants have a key role in allowing the following maturation. Glutathione has a key role in counteracting the apoptosis promoting effects of ROS [67]. An increased ROS in cumulus cells has been reported, in association with changes in tricarboxylic acid cycle and Nicotinamide-adenin-dinucleotide catabolism in follicular fluid of PCOS patients [68].

Infertility: it is well known that PCOS obese women have an increased time-to-pregnancy and a higher risk of miscarriage, and once again this could be mediated by OS [69]. Experimental models suggest that obesity induced by high-fat diet is associated with increased ROS production, antioxidant depletion, and abnormal distribution of mitochondria in oocytes [35].

Interplay between OS and LGI are very complex and often associated with reciprocal effects [70]. The role of obesity remains controversial, since it can induce both LGI and OS, and the phenotypes of obese PCOS patients are surely more severe than normal-weight PCOS subjects.

## 3. Role of Obesity: Pros

Obesity can induce both OS and LGI. Different studies showed increased levels of reactive species or by-products of oxidative damage in obesity [71,72,73,74]. OS has been linked to IR since it impairs glucose uptake in muscle and adipose tissue and reduces insulin secretion from pancreatic b-cells [53,54,55]. Increased levels of inflammatory cytokines in serum and altered lymphocyte function have been described [75,76,77] and related to complications such as diabetes, atherosclerosis, and steatohepatitis [78,79,80,81,82]. Obesity is considered paradigmatic, as a state of chronic LGI. Metabolic inflammation is characterized by an unbalanced expression of pro- and anti-inflammatory adipokines in the adipose tissue [83]. This unbalance affects insulin signaling, contributing to the development of IR and DM2 [84], in the so-called metabolic syndrome. Furthermore, IR and LGI have been recently associated with dysbiosis and altered intestinal permeability [85,86]. In this context lipocalin-2 (LCN2) may play an important role. Serum LCN2 levels are increased in obese patients [87,88], while discrepant results are reported on the correlation between LCN2 and IR indexes [89,90,91,92,93,94,95,96]. We showed increased levels in metabolic syndrome, but not in other conditions with IR such as growth hormone deficiency [97]. Finally, endogenous stimuli can be related to inflammation, including the stimulatory effects of free fatty acids on innate immune response [98,99].

Inflammatory markers related to obesity and not PCOS status per se include: TNFα, soluble type 2 TNF receptor, IL-6, and high sensitive CRP [100,101]. The increased central fat excess seems to be related to low-grade inflammation and IR [102]. TNFα is overexpressed in adipose tissue, induces IR [84] and probably the increase of the visceral adipose tissue is also the source in lean PCOS. 

Another proinflammatory cytokine is IL-18, which induces the production of T, in turn stimulating IL-6 synthesis [103]. 

Other markers related to abdominal obesity are increased plasminogen activator inhibitor-1 [104], increased angiotensin-renin system [105,106], and decreased adiponectin [107].

Interestingly, OS markers decrease after weight loss, induced by hypocaloric diet, natural antioxidants, and bariatric surgery [1,108,109,110].

## 4. Role of Obesity: Cons

A chronic androgen excess can induce obesity, influencing abdominal fat deposition [111]. Hyperandrogenism interacts with mitochondrial dysfunction above described; in experimental animals, IR can be induced by androgen overexposure via augmented ROS production. Pancreatic islets of rats treated with DHT showed lower mtDNA copies, oxygen consumption rate, and ATP production in comparison with untreated control animals [112]. 

Inflammatory markers, such as highly sensitive CRP, leukocytes count, neutrophil count and neutrophil/lymphocytes ratio (NLR) were found to be higher in PCOS, both obese and lean, compared with body weight-matched controls [113]. NLR showed a correlation with highly sensitive CRP; on the contrary, HOMA-index correlated with BMI. Therefore, the authors concluded that the condition of PCO rather than BMI induced this inflammatory state. No correlation was reported with androgens.

Few authors have studied PCOS women undergoing in vitro fertilization procedure in comparison with non-PCOS patients [114]. BMI of PCOS patients was positively correlated with leukocyte, neutrophil, lymphocyte, and mean platelet volume (*p* < 0.05), but negatively correlated with NLR and platelet/lymphocytes ratio (PRL, *p* < 0.05). Both NLR and PLR increased significantly in PCOS (*p* < 0.001). PLR increased significantly in NW-PCOS compared the NW-controls and obese PCOS (OB-PCOS). MPV values increased only in OB-PCOS subjects. The logistic regression analyses showed that MPV was the independent variable in PCOS to effect CPR (*p* = 0.000; OR 0.1; CI 0.06–0.2). Since NLR and PLR were significantly increased in all PCOS subjects compared to the BMI-matched controls, again the authors hypothesized that PCOS is a chronic inflammatory process independent of obesity. Despite PLR being decreased by adiposity, PLR increased in NW-PCOS. 

In agreement with these studies, Agakayak et al. investigated CRP, IL-6, TNFα, NLR in lean and obese PCOS subjects, supplemented with vitamin B12 and neopterin, a key molecule involved in immune response and useful for cardiovascular risk evaluation, released by macrophages of atherosclerotic plaques [115]. While CRP seemed to be linked to obesity, the other parameters were considered related to PCOS itself, including the lowering of B12, which has an anti-inflammatory action.

A model to investigate the relationships between obesity and inflammatory markers in PCOS is bariatric surgery. The decrease of CRP and adiponectin was present after such procedures, with slower effects in obese than in lean PCOS; the authors hypothesized a role for IR [116].

Another interesting approach is the proteomic analysis, which has been performed in NW-PCOS. Gene ontology analysis shows significant enrichment for terms related to inflammatory immune response, metabolism, and IGF-receptor signaling pathway. Circulating levels of IGF-1 and -2 and IGF binding protein-2, -3, and -4 are found to be lower in females with PCOS compared to healthy controls [117]. This is also confirmed in a study performed in follicular fluid, showing intrinsic abnormalities in PCOS patients, which were amplified by obesity [35].

## 5. The Underestimated Role of GH/IGF-1 Axis

The topic of GH-IGF axis in PCOS has been addressed in the nineties. Original data suggested lower GH secretion with normal IGF-1 values, but in a small and heterogeneous group of patients [118]. 

PCOS subjects have has lower levels of GH when hyperinsulinemic [119]; some investigations confirmed this datum, together with an inferior suppressive effect of GnRH antagonists [120]. Prelevic also showed low GH secretion only in obese PCOS [121].

Conflicting results concern GH levels, dynamics, and IGF-1 levels [121,122,123,124]. IGF-1 levels, together with other parameters of GH-dependent axis (IGFBP-1 and IGF-1/IGFBP-1 ratio) were not influenced by a treatment with metformin plus Vitamin D [125]; however, more than systemic levels, intraovaric IGF-1 levels could be more important for follicular growth. Interestingly, both IGF-I and IGF-II are lower in follicular fluid of PCOS women [126]. Immunohistochemical studies confirmed low expression of IGF-1 receptors in granulosa cells, associated with an increased expression in thecal-stromal cells [127] contributing to the complex endocrine picture of PCOS. Follicular IGF-1 was considered the only biomarker with significantly lower levels throughout a broad spectrum of women with fertility disorders [128].

Concerning our topic, IGF-1 could be a key molecule, not only in modulation of steroidogenic and follicular cell proliferation, but also in regulating the response to OS and LGI. We previously reviewed the role of GH-IGF-1 axis in the modulation of antioxidant systems [66,129].

Models of diabetes and hypertension suggest that IGF-1 can exert an anti-inflammatory role; higher IGF-1 levels protect against microvascular complications of hypertension and the development of type 2 diabetes mellitus [130]. IGF-1, together with IL, regulates levels of monocyte chemoattractant protein 1 (MCP-1) [131]. IGF-1, similarly to antioxidant glutathione, was lower in diabetic hypertensive patients [132]. An antiatherogenic effect of IGF-1 is reported, as supported by experiments in animals and humans, even if the mechanism remains still unclear [133,134,135]. Moreover, an effect on lipoxygenase with reduced lipid oxidation and foam cell formation has been demonstrated [80].

Interestingly, GH and IGF-1 can exert different effects on adipose tissue. GH has direct actions on mature adipocytes, inducing the release of free fatty acid and increased oxidation [136]; on the contrary, IGF-1 does not have effects on mature adipocytes, while they are produced by them. Adult mice with partial IGF-1 deficiency showed reduced expression of genes involved in lipid metabolism, cholesterol synthesis, and cholesterol transport [137]. Low IGF-1 is associated to IR, glucose intolerance, and diabetes [138]. Moreover, free IGF-1 levels are reduced in obesity and IGF-1 concentration was an independent factor associated with IR [139]. Finally, IGF-1 levels are inversely correlated with distribution of visceral adipose tissue [140,141,142,143,144]. IGF-1 can counteract the augmented ROS production induced by a high-fat diet [145].

Extrapolating these data to PCOS is still speculative; however, is it possible that low levels of IGF-1 in follicular fluid can contribute to the inflammatory and pro-oxidant environment.

## 6. Other Implications Due to Oxidative Stress

The associations of serum vitamin D levels alterations have been also examined in PCOS patients [146]. A key role in controlling inflammation and OS is clearly attributed to vitamin D [147]. In fact, it sustains normal mitochondrial functions [148,149]; active vitamin D, calcitriol, regulates generation of nuclear factor erythroid 2–related factor 2 (Nrf2) pathway involved in protection toward OS [150]. The expression of sirtuin 3 is modulated by metabolites of vitamin D [151]. Many antioxidants and anti-inflammatory cytokines are upregulated by vitamin D [152]; for example, glutathione-peroxidase and glutathione itself [153,154,155]. On these bases, vitamin D supplementation has been proposed in women affected by PCOS [156]. Studies on this topic are controversial [157,158,159]. A recent metanalysis demonstrated that vitamin D supplementation to PCOS patients had a beneficial effect on some markers (CRP, malondialdehyde, and total antioxidant capacity) but remained ineffective on others (nitric oxide and glutathione).

Despite this great interest, the other side of the coin is less investigated. Some links with other systems above discussed could be hypothesized. For example, among factors upregulated by vitamin D we can find Klotho, a protein produced in the kidney and involved in anti-aging mice phenotypes, also acting on antioxidants formation [160]; therefore, a reduced stimulation could have detrimental effects. 

Interestingly, glutathione stimulates vitamin D regulatory and glucose-metabolism genes [161]. In vitro glutathione deficiency induces OS, but also downregulation of vitamin D-binding protein (VDBP), vitamin D-25-hydroxylase, and vitamin D receptor, therefore interfering with vitamin D activation and activity. This observation represented the basis for supplementation of vitamin D plus L-cysteine (precursor of glutathione) with reduction of OS. The same study also showed in obese adolescents the same reduction in glutathione and vitamin D, together with increased IR, TNFα, and carbonyl-proteins. A positive correlation between glutathione status and 25-OH vitamin D was described. 

A model supporting the influence of OS on vitamin D metabolism was reported in a culture of bovine proximal tubule cells: an increase in OS, demonstrated by augmented lipid hydroperoxides, was related to reduced 1α- and 24-hydroxylases activities [162]. 

Recently, a peptide produced in the bone has arisen interest among researchers, the osteocalcin (OC) and its undercarboxylated form (u-OC). OC is one of the most represented protein in the bone matrix. Interestingly, OC was reported significantly increased in PCOS subjects compared to controls with a negative correlation against TNF-α. Moreover, different OC levels in subjects with PCOS are supposed to be responsible for the heterogeneity of this syndrome [163]. Contrastingly, a multicentric study, that evaluated 298 women with PCOS and 194 healthy controls, reported that OC levels were decreased in PCOS patients compared with controls [164]. Taking into account the precursor of osteocalcin, the undercarboxylated form, a study reported its regulatory role in energy metabolism by promoting insulin release and increase of adiponectin production [165]. The same author also reported that u-OC exhibits a pattern related to the weight-dependent manner in PCOS subjects. Thus, u-OC produced in the bone has an anabolic function in muscle and allows an increase in glucose uptake, suggesting a role of the skeleton as an endocrine organ in the pathogenesis of PCOS [165]. However, the essential mechanism of crosstalk between u-OC and androgen excess in PCOS remains incompletely understood. OS may represent a connection between the two since it has been demonstrated that OS can alter OC/u-OC ratio [166], and u-OC exerts protective effects in diabetes counteracting OS thus increasing insulin sensitivity [167]. It is still speculative that mutations in mitochondrial antioxidants enzyme can influence this equilibrium in the bone as demonstrated in some experimental models [168].

Finally, another interesting aspect linked to LGI could be immune dyscrasia. Immune mechanisms have been supposed to contribute to the development of PCOS [169]. We have previously shown increased levels of free light chains of immunoglobulins (FLCs) in PCOS [170]. FLCs and CRP are sentinel biomarkers of different aspects of the immune system, representing, respectively, adaptive and innate immunity. Alterations of Th1 and Th2 cells have been reported [171]. Augmented expression of CD4+/CD28 null lymphocytes, an aggressive subset with proinflammatory characteristics, has been reported [30]. Altered immunocompetent cells population have also been found in follicular fluid [172]. Our preliminary data, showing reduced levels of Ig1, Ig2, and Ig3 subclasses, together with high CH50 levels, suggesting an immune dyscrasia in PCOS (submitted). 

## 7. Conclusions

**Figure 2:**
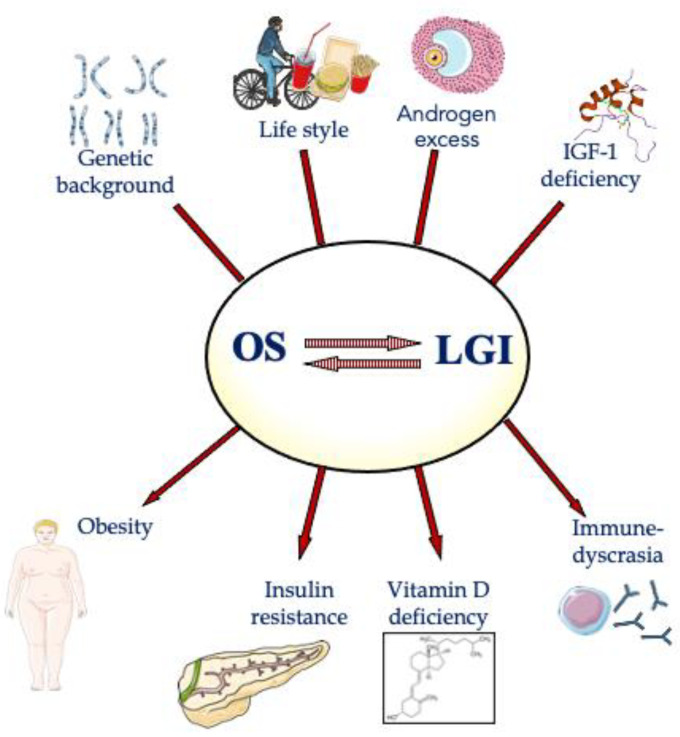
Figurative representation of the central role of oxidative stress (OS) and low-grade inflammation (LGI), with main etiological factors and consequences of their reciprocal interaction. Some illustration elements used in this figure have been kindly provided by the Servier. Servier Medical Art is licensed under a Creative Commons Attribution V.3.0 Unported License.

PCOS is a gynecological endocrine disorder reported in patients with heterogeneous clinical manifestations with different phenotypes. Moreover, environmental and genetic factors also have a role in the development of PCOS condition. Despite the complex scenario of this multifaceted disorder, OS and LGI, in a mutual reinforcing action, seem to be crucial in the pathogenesis of PCOS, both in normal weight and obese patients, with a severe picture in the last ones. They can be induced by the interaction from genetic background and lifestyle elements while hormonal events (hyperandrogenism and low IGF-1 at peripheral level) can be factor inducing or amplifying the OS-LGI status (Figure 2). In addition, considering the heterogeneity of the PCOS population, targeted therapies addressed to pathophysiological mechanisms have to be considered, even if at this moment scientific evidence to this approach is still lacking.

## Abbreviations

CRP	C-reactive protein
DHT	Dihydrotestosterone
FLCs	Free Light Chains of immunoglobulins
GH	Growth Hormone
HY	Hyperandrogenism
IGF-1	Insulin-like growth factor 1
HA IR	Hyperandrogenism Insulin Resistance
LCN2	Lipocalin-2
LGI	Low-grade inflammation
MCP-1	Monocytes chemoattractant protein 1
MNC	Mononuclear cell
NIH	National Insitute of Health
NLR	Neutrophils/Linfocytes ratio
Nrf2	Nuclear factor erythroid 2–related factor 2
NW-PCOS	Normal weight PCOS
OB-POCS	Obese PCOS
OC	Osteocalcin
OD	Ovulatory Disfunction
OS	Oxidative Stress
PCOM	Polycystic Ovary Morphology
PCOS	Polycystic Ovary Syndrome
ROS	Reactive Oxygen Species
T	Testosterone
TNF	Tumor Necrosis Factor
u-OC	Undercarboxylated Osteocalcin
